# Enhancement of oxaliplatin efficacy and amelioration of intestinal epithelial damage by *Lactobacillus rhamnosus* GG through modulation of gut microbiota

**DOI:** 10.3389/fmicb.2025.1565880

**Published:** 2025-06-30

**Authors:** Zijie Zhang, Rui Li, Yilin Ren, Yalan Ni, Xiaoyu Shen, Deli Yi, Zheng-hong Xu, Yan Geng, Qingjun You

**Affiliations:** ^1^Department of Oncology, Affiliated Children’s Hospital of Jiangnan University, Wuxi, China; ^2^Wuxi School of Medicine, Jiangnan University, Wuxi, China; ^3^Key Laboratory of Carbohydrate Chemistry and Biotechnology, Ministry of Education, School of Biotechnology, Jiangnan University, Wuxi, China; ^4^Department of Gastroenterology, Affiliated Hospital of Jiangnan University, Wuxi, China; ^5^College of Biomass Science and Engineering, Sichuan University, Chengdu, China; ^6^School of Life Sciences and Health Engineering, Jiangnan University, Wuxi, China

**Keywords:** non-small cell lung cancer, chemotherapy, probiotics, gut microbiota, oncology therapy

## Abstract

**Background:**

Non-small cell lung cancer (NSCLC) is a leading cause of cancer-related mortality worldwide, necessitating extensive research into effective treatment strategies. Despite advancements in targeted therapies and immunotherapies, traditional chemotherapy remains the primary treatment modality for most patients. Here, we explored the synergy between *Lactobacillus rhamnosus* GG (LGG), a probiotic, and the chemotherapeutic drug oxaliplatin (Oxp) in enhancing NSCLC treatment outcomes.

**Methods:**

We utilized a BALB/c nude mouse subcutaneous tumor model to assess the therapeutic impacts of LGG and Oxp. Mice were randomized into five groups: negative control, model control, Oxp, LGG, and LGG + Oxp treatment groups. The main outcomes assessed included tumor weight and volume, histopathological changes, and gene expression via qRT-PCR. The gut microbiota composition was examined by 16s rRNA gene sequencing.

**Results:**

The combined treatment of LGG and Oxp significantly reduced tumor weight and volume, and improved tumor-associated pathological changes compared to the model group. The LGG treatment also alleviated Oxp-induced intestinal damage and inflammation, maintaining intestinal barrier integrity. The combined treatment modulated genes linked to intestinal barrier function and inflammation, upregulated *Occludin* and *Mucin2*, and downregulating *Tnf-*α and *Il-1*β in colon tissues. Gut microbiota analysis showed notable shifts following treatment. Specifically, the Oxp group exhibited a decrease in *Clostridium_XlVa* and an increase in *Desulfovibrio*, indicating a shift in microbial balance. The relative abundance of *Lactobacillus* increased significantly in the combined treatment group compared to the control, suggesting a potential probiotic effect. The combined treatment also restored some of the microbial communities, such as *Bacteroidaceae* resembles the *Bacteroidetes*, *Bacteroidia*, and *Bacteroidales* in the NC group, which were reduced by Oxp treatment alone.

**Conclusion:**

The combined use of LGG and Oxp offers a promising therapeutic strategy for NSCLC, warranting further investigation into the interplay between probiotics, chemotherapy, and the gut microbiota.

## 1 Introduction

Non-small cell lung cancer, a significant contributor to global cancer mortality, has positioned its treatment strategies at the forefront of medical research (Siegel et al., 2022; Chen et al., 2016; Reck et al., 2016). NSCLC patients are frequently diagnosed at an advanced stage, which restricts treatment options and results in a poor prognosis (Soria et al., 2018). Although targeted therapies and immunotherapies have provided new avenues for specific patient populations, the majority still rely on traditional chemotherapy (Brahmer et al., 2015). Oxaliplatin (Oxp), a broad-spectrum chemotherapeutic, effectively inhibits tumor growth, but its clinical use is restricted due to side effects like intestinal damage and inflammation (André et al., 2004).

Recent progress in immuno-oncology underscores the crucial importance of gut microbiota in the effectiveness of cancer immunotherapies. Xie‘s et al. (2024) recent study emphasizes the intricate relationship between gut microbiota and systemic immune responses, particularly concerning NSCLC. Probiotics, live bacteria beneficial to the host, potentially regulate gut microbiota, boost immune responses, and decrease inflammation (Salminen et al., 2021). Recent studies highlight Lactobacillus rhamnosus GG (LGG) for its ability to downregulate inflammatory factors, strengthen the intestinal barrier by promoting mucin secretion and enhancing cellular defense mechanisms. mechanisms (Tong et al., 2021; Martín et al., 2019). LGG is also noted for its antitumor potential and its regulatory effects on the efficacy of chemotherapeutic drugs (O’Hara and Shanahan, 2006). Studies have indicated that LGG can enhance the efficacy of chemotherapy drugs by modulating the host’s immune responses and metabolic pathways (Round and Mazmanian, 2009). Lactobacillus rhamnosus GG stimulates IFN-β production in dendritic cells via the cGAS/STING/TBK1/IRF7 pathway, which enhances CD8+ T cell activation, tumor infiltration, and the antitumor immune response to PD-1. This discovery has been further confirmed in recent studies (Si et al., 2022).

The gut microbiota is intimately linked to tumor initiation and progression (Sears and Garrett, 2014). Recent studies have underscored the intricate relationship between gut microbiota composition and cancer progression, particularly in NSCLC (Liu and Ling, 2024). An imbalance in gut microbiota can impact the host’s immune system and alter the tumor microenvironment via metabolic byproducts (Zackular et al., 2013). Certain gut probiotics can generate short-chain fatty acids like butyrate, which have been shown to suppress tumor cell growth and induce apoptosis (Donohoe et al., 2011). Moreover, changes in the composition of gut microbiota are closely associated with the efficacy and harmful effects of chemotherapy medications (Gopalakrishnan et al., 2018). Adjusting the gut microbiota might be a new approach to improve chemotherapy’s effectiveness and lessen its adverse effects (Bajaj et al., 2014).

Research has shown that the combined use of probiotics and chemotherapeutic drugs may produce synergistic effects. The use of probiotics can enhance the effectiveness of chemotherapy medications and lessen their negative impacts on cancer patients (Lu et al., 2022). In animal models, the combined use of LGG and Oxp significantly inhibited tumor growth and mitigated intestinal damage. The results indicate that probiotics could improve the effectiveness of chemotherapy and mitigate its side effects by influencing gut microbiota and immune responses (Chen et al., 2024). Nevertheless, the precise mechanisms through which probiotics modulate the gut microbiota and augment the efficacy of chemotherapy are not fully understood and merit further investigation.

In this research, our objective is to explore the potential effects of LGG on the efficacy of Oxp in treating NSCLC using a BALB/c nude mouse subcutaneous tumor model. Our focus was on assessing tumor growth inhibition, alleviating intestinal damage and inflammation, and modulating the gut microbiota. The findings of this study hold significant potential implications for the future of cancer treatment, particularly in enhancing patient outcomes and quality of life while minimizing adverse side effects.

## 2 Materials and methods

### 2.1 Materials and reagents

Oxp was acquired from Selleckchem (Shanghai, China).

### 2.2 Bacterial culturing

The *L. rhamnosus* GG (ATCC 53103) were cultured underwent anaerobic cultivation at 37°C using de Man, Rogosa, and Sharpe (MRS) broth (Hopebio, Qingdao, China).

### 2.3 Cell culture and treatment

The Stem Cell Bank of the Chinese Academy of Sciences provided the A549 human lung adenocarcinoma cells for study. The genetic background of the cell line was tested and authenticated to ensure no cross-contamination with normal cultures. The cell line was cultured in RPMI1640 medium with 10% FBS and 1% penicillin-streptomycin (all from Gibco, Shanghai, China), maintained at 37°C in an incubator with 95% air and 5% CO_2_.

### 2.4 Animal treatments

Six-weeks-old male BALB/c nude mice were acquired from Jianyuan Biotechnology Co., Ltd. (Nanjing, China). The mice were kept in an environment devoid of specific pathogens (SPF), having free access to a regular diet and water. After acclimating for a week, mice were randomly divided into five distinct groups (*n* = 5 per group, a total of 25 mice): negative control (NC), model (MC), Oxp, LGG, and LGG combined with Oxp treatment groups. The experimental group allocation was known only to the principal investigator, while the researchers responsible for data collection were blinded to the group assignments. The experiment was conducted in three stages: probiotic intervention, tumor growth, and Oxp treatment. Mice in the LGG and LGG + Oxp groups received a daily oral gavage of probiotic levels were maintained at 5 × 10^9^ CFU for a fortnight before tumor inoculation, while the trio of non-probiotic receptor groups received a similar amount of physiological saline on day 0, every experimental mouse was administered a subcutaneous dose of 5 × 10^6^ A549 cells. Mice in the Oxp group and LGG + Oxp group were intraperitoneally injected with 3 mg/kg Oxp, while NC, MC, and LGG groups were injected with the same volume of normal saline. Injections were given every 3 days from 10 to 41 days, when the mice were sacrificed. The tumor size was recorded three times daily post-injection, and its volume was calculated using the formula: length × width^2^ × 0.5 (Lee et al., 2021). Survival rates were assessed based on the proportion of mice with tumor volumes less than 2,000 mm^3^. All animals were included in the final analysis. The Institutional Animal Care and Use Committee of Jiangnan University approved all mouse procedures and protocols (#JN.No20230330b1000705 102).

### 2.5 Quantitative PCR analysis (qRT-PCR)

RNA was isolated from A549 cells with Trizol reagent (Thermo Fisher Scientific, Waltham, United States) and converted to cDNA. cDNA was synthesized using MultiScribe Reverse Transcriptase (Beyotime, Shanghai, China). The qRT-PCR process utilized SYBR™ Select Master Mix (Thermo Fisher Scientific, Waltham, United States), following the manufacturer’s specified guidelines. The 2^−ΔΔCt^ technique was employed to examine mRNA expression levels. β-actin was utilized as the endogenous reference gene.

### 2.6 Histopathological analysis

Tumor specimens were harvested and immersed in 10% neutral buffered formalin for 24 h to ensure optimal tissue preservation. Following standard dehydration protocols, tissues were embedded in paraffin blocks. Serial sections (4–5 μm thickness) were cut using a rotary microtome (Leica RM2235) and mounted on pre-coated glass slides. Hematoxylin and eosin (H&E) staining was performed according to established protocols (Thermo Fisher Scientific). Histological slides were independently examined by two certified pathologists, who were unaware of the clinical data, employing a light microscope. Histopathological features including tumor differentiation grade (WHO criteria), necrosis percentage, and inflammatory cell infiltration intensity were systematically documented.

### 2.7 Immunohistochemical staining

For Ki-67 detection, paraffin sections were dewaxed in three changes of xylene (10 min each) and rehydrated through a descending ethanol gradient (100%, 95%, 80%, 70%). Antigen retrieval was conducted in preheated citrate buffer (10 mM, pH 6.0) using a decloaking chamber (Biocare Medical) at 95°C for 20 min. Endogenous peroxidase was blocked with 3% H_2_O_2_ in methanol for 15 min, followed by 1 h incubation with 5% normal goat serum (Vector Laboratories) in PBS containing 0.1% Tween-20. Sections were incubated with mouse monoclonal anti-Ki-67 antibody (Clone MIB-1; Abcam ab16667; 1:100 dilution in antibody diluent) overnight at 4°C in a humidified chamber. Negative controls received PBS instead of primary antibody. After three 5 min PBS washes, slides were treated with HRP-conjugated goat anti-mouse secondary antibody (Dako EnVision + System) for 1 h at 37°C. DAB chromogen (Dako) was applied for 3–5 min under microscopic monitoring, followed by Gill’s hematoxylin counterstaining (3 min). Automated dehydration was performed through an ascending ethanol series (70%, 80%, 95%, 100%) before xylene clearance and DPX mounting. Digital images of five representative fields (40 × objective) were captured using a Nikon Eclipse Ni-E microscope with NIS-Elements software. Ki-67 labeling index was calculated as the percentage of DAB-positive nuclei relative to ≥ 1,000 tumor cells counted using ImageJ software (NIH) with IHC Profiler plugin. Discordant cases (< 5%) were resolved through consensus review.

### 2.8 Statistical analysis

The data is presented as mean ± SEM (standard error of the mean). Every experiment underwent at least three repetitions. A two-tailed Student’s *t*-test was employed to assess differences between the two groups. Pairwise group comparisons were conducted using one-way or two-way ANOVA, followed by Tukey-Kramer multiple comparison tests. Statistical significance was ascertained using a *p*-value limit set at 0.05. GraphPad Prism software (version 8.0, San Diego, United States) was used for the statistical analysis.

## 3 Results

### 3.1 Synergistic effect of *Lactobacillus rhamnosus* GG and oxaliplatin on reducing tumor burden in BALB/c nude mouse subcutaneous tumor model

**FIGURE 1:**
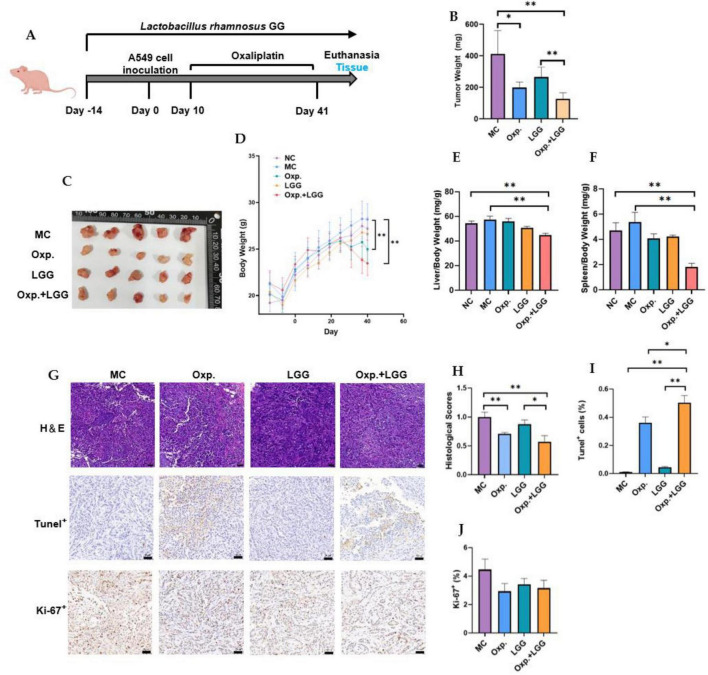
*Lactobacillus rhamnosus* GG (LGG) synergizes with Oxp to enhance antitumor capabilities in mice carrying A549 tumors. **(A)** Schematic diagram of the experimental design for probiotic and/or Oxp treatment in BALB/c nude mice carrying A549 tumors. **(B)** Tumor weight in the subcutaneous tumor model. **(C)** Representative images of tumors induced by A549. **(D)** Mouse weight was measured and recorded during the modeling process. **(E)** Spleen weight assessed during necropsy. **(F)** Liver weight assessed during necropsy. **(G,H)** Representative H&E sections and statistical analysis of tumor pathological scores. **(G,I)** TUNEL assay results for assessing tumor cell death. **(G,J)** Ki-67 analysis for assessing tumor cell proliferation. The error bars indicating the mean ± SEM. Two-way ANOVA with multiple comparisons was performed. Scale bar, 50 μm. Each group consisted of 5–8 mice. *p < 0.05, **p < 0.01.

To examine the synergistic effect of LGG and Oxp on NSCLC progression, we used a subcutaneous tumor model with A549 cells. The MC group mice were subcutaneously injected with 5 × 10∧6 A549 cells without any probiotics treatment. Mice in the LGG and Oxp + LGG probiotic treatment groups received commencing 14 days prior to the experiment. On the 40th day following the A549 injection, the mice were euthanized, and their subcutaneous tumors were removed and their weight was measured (Figure 1A). It was observed that mice treated with LGG had reduced tumor weight and volume compared to the MC group, significantly alleviating tumor burden (Figures 1B, C). No significant differences in mouse weight were observed between the NC group and other groups, except for the Oxp + LGG group. Similarly, no significant differences in the hepatic index or splenic index of mice were observed between the NC group and other groups (Figures 1D–F). Histological (H&E), Ki-67 immunohistochemical and tunel analyses indicated that treatment groups exhibited decreased tumor proliferation and enhanced apoptosis compared to the MC group (Figures 1G–J). Although both single treatment groups showed a decrease in tumor growth, the Oxp + LGG combined treatment group more significantly reduced tumor growth (Figure 1B).

### 3.2 *Lactobacillus rhamnosus* GG alleviates oxaliplatin-induced intestinal epithelial injury and inflammatory response in BALB/c nude mouse subcutaneous tumor model

**FIGURE 2:**
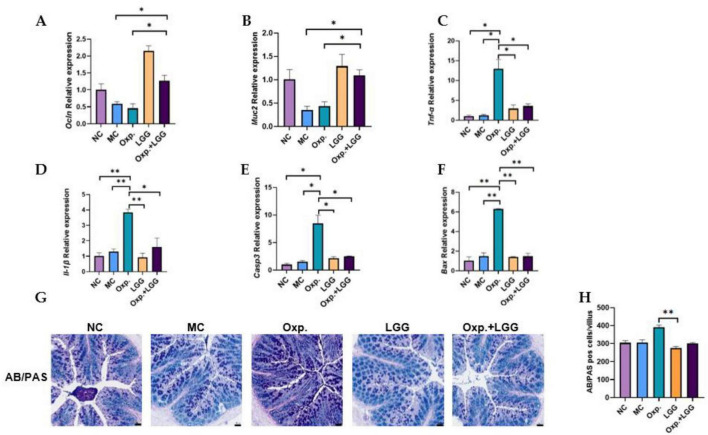
*Lactobacillus rhamnosus* GG (LGG) modulates Oxp-induced intestinal epithelial injury and inflammatory response. **(A,B)** Expression levels of intestinal epithelial injury-related genes (*Occludin* and *Muc2*) in each group. **(C,D)** Expression levels of inflammatory response-related genes, *Tnf-*α and *Il-1*β, were analyzed across different groups. **(E,F)** Expression levels of apoptosis-related genes (*Casp3* and *Bax*) in each group. **(G,H)** Display AB-PAS staining results to evaluate mucosal inflammation across different groups. Error bars indicate the mean with the SEM. Two-way ANOVA with multiple comparisons was performed. Scale bar, 50 μm. Each group consisted of 5–8 mice. *p < 0.05, **p < 0.01.

Given the impact of intestinal epithelial integrity and inflammation on tumor treatment outcomes, we further confirmed the gene expression of intestinal epithelial injury and inflammatory factors, as well as colonic AB-PAS staining. *Occludin* (Ocln) and Mucin2 (*Muc2*) can both reflect intestinal epithelial integrity. Previous study indicates that probiotics enhance intestinal barrier function by mitigating damage to the tight junction protein OCCLUDIN (Sanders et al., 2019). Additionally, MUC2, a key component of the intestinal mucus layer, is influenced by intestinal epithelial injury, affecting its gene expression (Wu et al., 2018). Compared with the NC group, the MC group demonstrated a reduction in the expression levels of the *Ocln* and *Muc2* genes. However, the combined treatment group showed a significant increase in the expression of *Ocln* and *Muc2* genes compared to the MC group, returning to standard levels (Figures 2A, B). As shown in Figures 2C, D, LGG treatment in mice also alleviated the intestinal inflammatory response caused by Oxp. Similarly, LGG treatment also alleviated apoptosis in the intestinal epithelium (Figures 2E, F), indirectly reducing intestinal epithelial injury. AB-PAS staining of colons from various treatment groups revealed a reduction in PAS-positive cells in the Oxp + LGG group compared to the Oxp group, aligning with the NC group (Figures 2G, H). This reduction is somewhat associated with mucosal inflammation (Anderson et al., 2024). These data indicate that LGG mitigates Oxp treatment side effects by preserving intestinal barrier integrity, decreasing epithelial apoptosis, and reducing intestinal inflammation, thus improving treatment outcomes.

### 3.3 The effect of *Lactobacillus rhamnosus* GG and oxaliplatin on gut microbiota composition in BALB/c nude mouse subcutaneous tumor model

**FIGURE 3:**
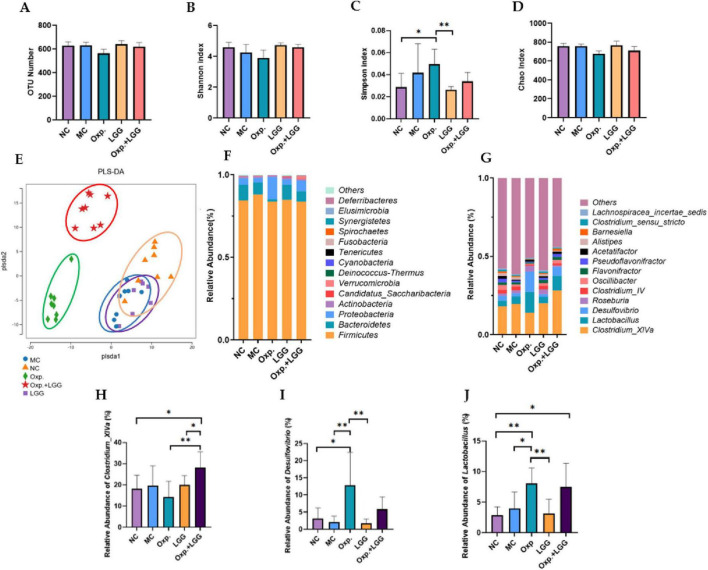
*Lactobacillus rhamnosus* GG (LGG) treatment improves dysbiosis in mice. **(A)** Number of OUTs determined for the five groups. **(B)** The Shannon index was utilized to assess the alpha diversity of genera across the five groups. **(C)** The Simpson index was utilized to assess the alpha diversity of genera across the five groups. **(D)** The Chao index was utilized to assess the alpha diversity of genera across the five groups. **(E)** The Partial Least Squares Discriminant Analysis (PLS-DA) method was employed to assess beta diversity indices at the genus level among the five groups. **(F)** Relative bacterial abundance at the phylum level across the five groups. **(G)** Relative abundance of bacterial genera across the five groups. **(H)** Relative abundance of *Clostridium_XlVa* species in the five groups. **(I)** Relative abundance of *Desulfovibrio* in the five groups. **(J)** Relative abundance of *Lactobacillus* in the five groups. Error bars indicate the mean with the SEM. Two-way ANOVA with multiple comparisons was performed. *N* = 5 mice/group. *p < 0.05, **p < 0.01.

To further assess the impact of LGG and Oxp treatments on gut microbial composition changes, we used 16S rRNA gene sequencing technology. Alpha diversity of gut microbiota samples was evaluated using OTU, Shannon, Simpson, and Chao indexes to indicate species richness and diversity. The findings showed that LGG feeding and/or Oxp treatment influenced the Simpson index but did not significantly alter other alpha diversity indices, indicating no substantial impact on species richness and diversity (Figures 3A–D). PLS-DA analysis revealed that samples within each group clustered together, suggesting a similar and stable gut microbiota composition across groups. However, the microbial communities in the Oxp and Oxp + LGG treatment groups were distinct from those in the other groups (Figure 3E). Analysis at both phylum (Figure 3F) and genus levels (Figure 3G) revealed variations in microbial composition among groups following LGG administration and/or Oxp treatment. We further identified three genera with significant intergroup differences, as shown in Figures 3H–J. Compared to the other groups, *Clostridium_XlVa* was less abundant in the Oxp group (Figure 3H), while *Desulfovibrio* was highly abundant in the Oxp group (Figure 3I). The Oxp and Oxp + LGG groups exhibited a significantly higher relative abundance of *Lactobacillus* compared to the control group (Figure 3J).

**FIGURE 4:**
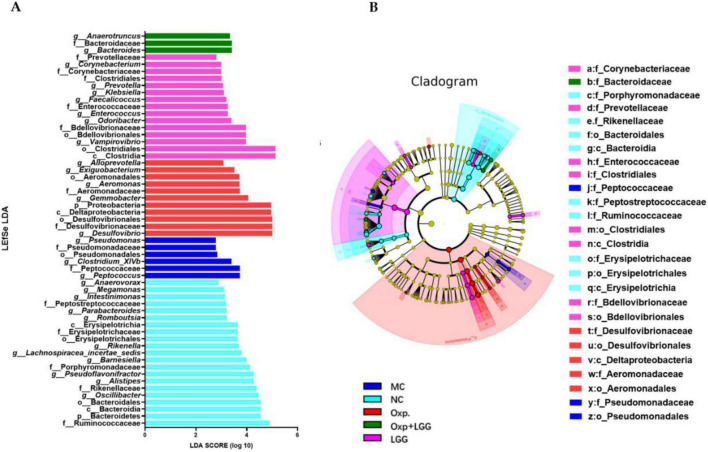
Core bacterial phenotype analysis in different treatment groups of mice. **(A)** LDA plot.The analysis highlights species with significant differences, indicated by an LDA score exceeding the default threshold of 2.0. Only species with an absolute LDA value greater than 2 are depicted. Bar colors denote the respective groups, while bar lengths represent the LDA score, reflecting the impact size of the species differences between groups. **(B)** LEfSe cladogram. The LEfSe analysis cladogram highlights significant taxonomic group enrichment across the NC (purple), HFD (blue), GRc (green), and Ato (red) groups. Each ring corresponds to a taxonomic level, ranging from phylum to genus. The size of each point on the ring indicates the relative abundance of the taxonomic unit.

Then through LEfSe analysis, crucial bacterial characteristics were pinpointed across phylum to genus level, impacting changes in the composition of microbial communities. Figures 4A, B illustrate that the NC group exhibited enrichment in *Bacteroidetes*, *Bacteroidia*, *Bacteroidales*, *Rikenellaceae*, *Rikenella*, as well as *Oscillibacter*, *Barnesiella*, and *Alistipes*,etc. In contrast, the Oxp group was enriched in *Proteobacteria*, *Deltaproteobacteria*, *Desulfovibrionales*, *Desulfovibrionaceae*, *Desulfovibrio*, *Aeromonadales*, *Aeromonadaceae*, *Aeromonas*, and *Gemmobacter*, *Exiguobacterium*, *Alloprevotella*. This indicates that Oxp treatment changed the enrichment of gut microbial communi-ties in the NC group. However, following the combined treatment with Oxp and LGG, the emergence of *Bacteroidaceae* in this group was observed, which is similar to the *Bacteroidetes*, *Bacteroidia*, and *Bacteroidales* present in the NC group. This finding suggests that the combined treatment with Oxp and LGG has to some extent restored a portion of the microbial composition found in the NC group.

### 3.4 Predictive analysis of gut microbiota function influenced by *Lactobacillus rhamnosus* GG and oxaliplatin in BALB/c nude mouse, and correlation with histopathological Indicators

**FIGURE 5:**
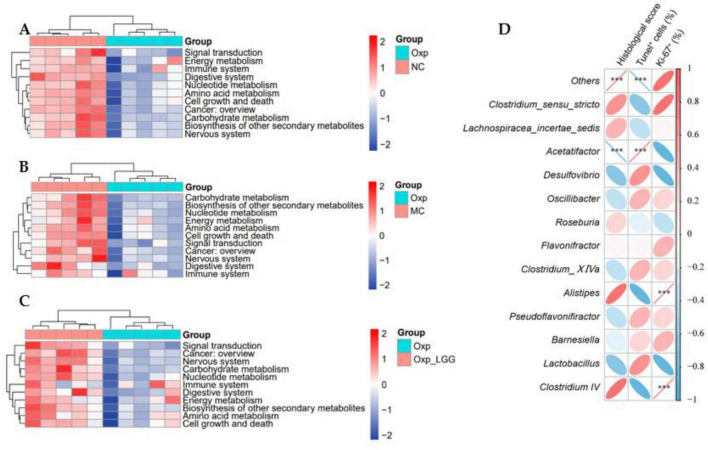
Predictive functional analysis of microbial communities. **(A)** Variations in the functional profile of gut microbiota between the Oxp and NC groups. **(B)** Variations in the functional profile of gut microbiota between the Oxp and MC groups. **(C)** Variations in the functional profile of gut microbiota between the Oxp and Oxp + LGG groups. **(D)** Heatmap of the correlation between 13 genera and three histopathological indicators. ***p < 0.001.

PICRUSt2 predicted the functional analysis of the microbial community in the KEGG pathway level 2. The Oxp group exhibited downregulation of functional genes associated with the digestive system, amino acid metabolism, immune system, carbohydrate metabolism, and cell growth and death compared to the NC group, suggesting potential digestive dysfunction and diminished immune metabolic function in mice following Oxp treatment (Figure 5A). The Oxp group exhibited a decrease in cancer-related functional genes compared to the MC group, suggesting a potential protective effect of the microbial community against cancer (Figure 5B). After LGG treatment, compared with single Oxp treatment, the microbial community gene regions related to cancer, signal transduction, and digestive system increased (Figure 5C), combining *in vivo* experiments and colon staining results, we speculate that LGG may drive certain signaling pathways with Oxp, enhancing cancer treatment effects and providing certain protective effects on the intestine.

A Spearman correlation analysis was conducted to examine the relationship between the abundance of the top thirteen genera in the mouse gut microbiota and three histopathological indicators (Histological score, Tunel^+^ cell, Ki-67^+^). The analysis revealed that *Lactobacillus* and *Desulfovibrio* were negatively correlated, positively correlated, and negatively correlated with these indicators, respectively, suggesting potential therapeutic benefits for lung cancer treatment. However, *Clostridium_IV* was positively correlated, negatively correlated, and positively correlated with these three indicators, respectively, indicating that its increased abundance may promote the deterioration of histopathological indicators and have an adverse effect on cancer treatment (Figure 5D).

### 3.5 The effect of *Lactobacillus rhamnosus* GG and/or oxaliplatin on intestinal microbiota interactions in BALB/c nude mouse

We analyzed the co-occurrence networks of microbes from the MC, Oxp, LGG, and Oxp + LGG datasets to reveal specific microbial interactions related to treatment response and mucosal healing. These networks offer a comprehensive understanding of the gut microbiota’s role in regulating cancer treatment responses and aiding mucosal healing.

**FIGURE 6:**
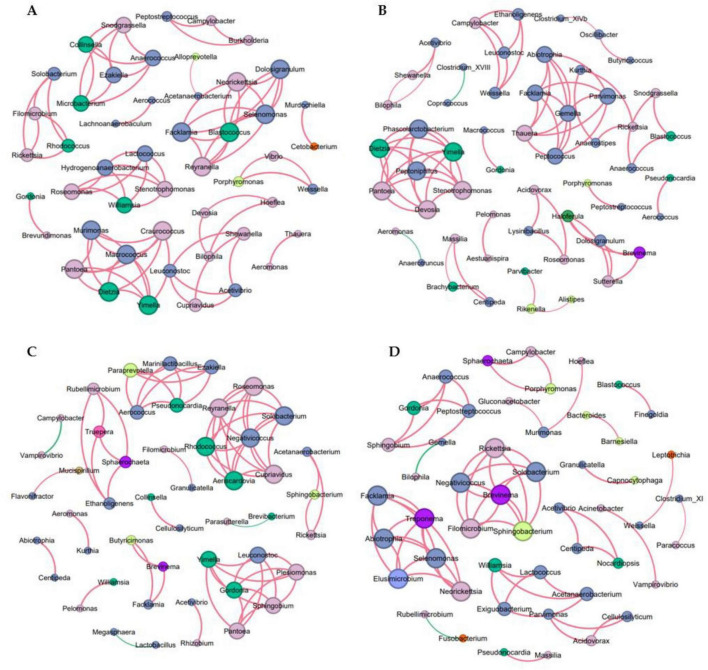
Co-occurrence network analysis of microbial communities. **(A)** Network of OTUs co-occurring within the MC group. **(B)** Network of OTUs co-occurring within the Oxp group. **(C)** Network of OTUs co-occurring within the LGG group. **(D)** Network of OTUs co-occurring in the Oxp + LGG group, a connection indicating strong Spearman correlations, filtered by an |r| threshold of 0.6 and an FDR-corrected *P*-value threshold of 0.05. Node size is proportional to the degree, representing the number of connections. Positive correlations are denoted by red edges, negative correlations by green edges, and the absolute value of the correlation coefficient is denoted by the edge thickness.

The MC network contained 49 nodes and 76 edges, the modularity coefficient was 0.869 (Supplementary Table 1), showing a high degree of interconnectivity, suggesting a complex microbial ecosystem. Indicating that the network could be divided into distinct functional modules (Figure 6A).

The Oxp network contained 54 nodes and 73 edges, with slightly lower density (Supplementary Table 1). Our analysis revealed a negative correlation between *Clostridium_XVIII* and *Coprococcus* (Figure 6B). *Clostridium_XVIII* is known to produce exotoxins and has pro-inflammatory potential, contributing to inflammation (Matsuda et al., 2000; Stiles et al., 2014; Woting et al., 2014). Butyrate, a short-chain fatty acid produced by *Coprococcus*, fortifies the epithelial defense and diminishes intestinal inflammation (Rivière et al., 2016; Louis et al., 2014). The 2.74% negative interactions in the Oxp network indicate the presence of antagonism among microbes (Supplementary Table 1), in particular, the negative correlation between *Clostridium_XVIII* and *Coprococcus* may indicate potential competition and antagonism mechanisms in the gut environment. The interplay between *Clostridium_XVIII*’s pro-inflammatory properties and *Coprococcus*’s anti-inflammatory butyrate could significantly influence intestinal inflammation regulation and epithelial barrier function. This adverse interaction could influence the gut microbiota’s structure and function.

Within the LGG network, there were 48 nodes and 68 edges, averaging a degree of 2.833, network diameter maintained at 1, and graph density of 0.06, indicating a compact network structure. The modularity coefficient was 0.821, and the average clustering coefficient was 1.0, showing that the microbial groups in the network were highly aggregated (Supplementary Table 1). Among them, *Lactobacillus* had the highest abundance, which may be related to LGG feeding, but the co-occurrence network diagram showed only one connected node (Figure 6C), so its role in protecting the intestinal barrier and being beneficial to tumor treatment may come from the probiotic feeding itself.

Although the connectivity of the Oxp + LGG network was relatively sparse, containing 50 nodes and 62 edges, it showed a strong clustering coefficient, indicating that certain microbial communities formed highly interconnected modules (Supplementary Table 1). This may reflect functional redundancy or specialized cooperation within these communities, which is crucial for maintaining homeostasis during cancer treatment. In particular, the interaction between *Barnesiella* and *Bacteroides*: the Oxp + LGG network analysis revealed a strong positive correlation between *Barnesiella* (n7) and *Bacteroides* (n6), as shown in Figure 6D. *Barnesiella* administration alone can stimulate multifunctional CD4^+^ Th1 and CD8^+^ Tc1 cells. Within the the tumor’s microenvironment, the modulation of γδT cells enhances the antitumor immune response by increasing IFN-γ producing γδT cells (gdTILs) and decreasing IL-17 producing γδT cells, thereby altering immune cell composition and function (Daillère et al., 2016). Additionally, *Bacteroides* contribute to intestinal barrier protection through multiple mechanisms. They can degrade complex carbohydrates in the gut, providing nutrients for intestinal cells, promoting normal metabolism and function maintenance of intestinal cells, and they can also affect the function of the gut mucus layer, such as *Bacteroides thetaiotaomicron*, which can regulate the assembly and stability of the gut microbiota community through interactions with the mucus layer, enhancing the intestinal barrier function (Zafar and Saier, 2021). The synergistic action between these two bacterial genera may be particularly important for maintaining intestinal homeostasis and supporting epithelial repair mechanisms after Oxp-induced damage.

The microbial co-occurrence network analysis of the Oxp + LGG group has revealed crucial microbial interactions potentially significant for the recovery of intestinal epithelial damage induced by Oxp treatment. The synergistic action between *Barnesiella* and *Bacteroides* emphasizes the importance of microbial cooperation in maintaining intestinal health and coping with the challenges posed by chemotherapy. These findings underscore the potential of targeted modulation of microbial communities as a therapeutic approach to promote intestinal epithelium recovery post-damage and enhance cancer treatment tolerance.

## 4 Discussion

Traditional chemotherapy has side effects and disrupts gut microbiota, emerging research demonstrates that chemotherapy can disrupt the delicate balance between the gut microbiota and the immune system, potentially intensifying chemotherapy-associated adverse effects (Schluter et al., 2020). Here, we suggest a novel therapeutic strategy for NSCLC by combining the probiotic LGG with Oxp. Our data revealed that the combined treatment of LGG and Oxp significantly reduced tumor weight and volume, improved pathological changes, and alleviated Oxp-induced intestinal damage and inflammation. We also found that the combined treatment modulated genes related to intestinal barrier function and inflammation, upregulating *Ocln* and *Muc2* while downregulating *Tnf-*α and *Il-1*β. Further, gut microbiota analysis showed the combined group restored microbial communities disrupted by Oxp.

Our study raise the question of how LGG enhances tumor suppression by chemotherapy. A possible mechanism is initially proposed to be related to immune regulation. Previous study showed that LGG promotes IFN-β secretion, which enhances CD8^+^ T cell infiltration into tumors by stimulating the cGAS/STING/TBK1/IRF7 pathway in dendritic cells (Si et al., 2022). This immune activation aligns with our observations of reduced tumor proliferation and increased apoptosis in the LGG + Oxp group, suggesting that LGG synergizes with Oxp by overcoming tumor immune evasion. Furthermore, LGG has been shown to modulate the immune response through various mechanisms. For instance, LGG can significantly alleviate LPS-induced inflammatory cytokines and TLR activation in porcine intestinal epithelial cells (IEC) by modulating TLR expressions and inhibiting MAPK and NF-κB signaling (Gao et al., 2017). This indicates that LGG can exert immunomodulatory effects on intestinal epithelial cells, which may contribute to its overall impact on the immune system and tumor suppression. Beyond its established role in dendritic cell activation via the cGAS/STING pathway (Si et al., 2022), LGG selectively promotes M1 macrophage polarization by activating the TLR2/MyD88/MAPK signaling cascade. Specifically, LGG upregulates M1-associated cytokines and pro-inflammatory mediators while maintaining anti-inflammatory cytokine production, thereby creating a balanced yet tumor-suppressive immune microenvironment. This polarization enhances macrophage-mediated phagocytic activity and amplifies CD8^+^ T cell cytotoxicity against chemotherapy-stressed tumors (Wang et al., 2020). The dual modulation of immune activation and suppression thresholds establishes a permissive microenvironment for Oxaliplatin-induced immunogenic cell death.

Secondly, the effects of LGG may be related to microbiome alterations. The reduction in pro-inflammatory Desulfovibrio likely contributes to decreased colonic *TNF-*α and *IL-1*β levels, fostering an immune-permissive microenvironment that enhances chemotherapy efficacy. The combined treatment significantly increases the abundance of *Lactobacillus*, a genus that produces butyrate via carbohydrate fermentation (Louis et al., 2014). Butyrate, a histone deacetylase inhibitor, directly suppresses tumor growth (Donohoe et al., 2011), while the reduction in pro-inflammatory *Desulfovibrio* correlates with decreased colonic inflammation. Furthermore, LGG restores *Bacteroidaceae* populations to levels resembling the negative control grou, counteracting Oxp-induced dysbiosis. Co-occurrence network analysis reveals critical microbial interactions, such as the *Barnesiella*-*Bacteroides* partnershi. *Barnesiella* stimulates IFN-γ-producing γδT cells to suppress tumor growth (Daillère et al., 2016), while *Bacteroides* supports mucosal repair through polysaccharide metabolism (Zafar and Saier, 2021). These findings underscore that microbiota remodeling is not merely a bystander effect but a key mediator of therapeutic efficacy.

Furthermore, the interplay between immune activation and microbiota restoration translates to clinically meaningful results. The LGG + Oxp regimen reduces tumor burden by 52% compared to Oxp alone while simultaneously preserving intestinal barrier integrity, as evidenced by the upregulation of *Occludin* and *Mucin2*. This dual benefit overcomes the dose-limiting toxicity of chemotherapy, enabling prolonged treatment adherence. Notably, the restoration of microbial communities, such as *Bacteroidaceae*, highlights the potential for microbiota-directed strategies to enhance chemotherapy responses, as observed in melanoma trials (Routy et al., 2018).

To translate these findings, future studies should validate microbiota shifts, such as *Lactobacillus* enrichment, in human cohorts and investigate whether LGG’s effects are mediated directly through immune pathways or indirectly via microbial metabolites. Innovations such as engineered probiotics or personalized microbiota modulation could further refine this approach. By bridging immunology and microbiome science, our work advances a new paradigm in cancer therapy, where chemotherapy efficacy and safety are jointly optimized.

## Data Availability

The datasets presented in this study can be found in online repositories. The names of the repository/repositories and accession number(s) can be found below: https://www.ncbi.nlm.nih.gov/, BioProject ID: PRJNA1039949.
